# Acute exercise inhibits gastric emptying of liquids in rats: influence of the NO-cGMP pathway

**DOI:** 10.1590/1414-431X20187541

**Published:** 2018-10-04

**Authors:** A.K.M. Cavalcante, R.C.L. Siqueira, V.N. Feitosa, C.R. de Andrade, A.A. Santos, M.T.B. Silva

**Affiliations:** 1 Programa de Pós-Graduação em Ciências Biológicas/Biotecnologia, Centro Universitário UNINTA, Sobral, CE, Brasil; 2 Programa de Pós-Graduação em Ciências Médicas, Universidade Federal do Ceará, Fortaleza, CE, Brasil; 3 Departmento de Fisiologia e Farmacologia, Faculdade de Medicina, Universidade Federal do Ceará, Fortaleza, CE, Brasil; 4 Laboratorio de Pesquisa Translacional, Centro Universitário Christus (UniChristus), Fortaleza, CE, Brasil; 5 Departmento de Educação Física, Universidade Federal do Piauí, Teresina, PI, Brasil

**Keywords:** Acute exercise, Bioelectrical impedance, Gastric motility, High-intensity exercise

## Abstract

We previously found that acute exercise inhibited the gastric emptying of liquid in awake rats by causing an acid-base imbalance. In the present study, we investigated the involvement of the nitric oxide-cyclic guanosine monophosphate (NO-cGMP) pathway, vasoactive intestinal peptide (VIP), and corticotropin-releasing factor (CRF) peptide in this phenomenon. Male rats were divided into exercise or sedentary group and were subjected to a 15-min swim session against a load (2.5 or 5% b.w.). The rate of gastric emptying was evaluated after 5, 10, or 20 min postprandially. Separate groups of rats were treated with vehicle (0.9% NaCl, 0.1 mL/100 g, *ip*) or one of the following agents: atropine (1.0 mg/kg, *ip*), the NO non-selective inhibitor Nω-nitro-L-arginine methyl ester hydrochloride (L-NAME; 10.0 mg/kg, *ip*), or the selective cGMP inhibitor 1H-(1,2,4)oxadiazole[4,3-a]quinoxalin-1-one (ODQ; 5.0 mg/kg, *ip*), the i-NOS non-specific inhibitor (aminoguanidine; 10.0 mg/kg, *ip*), the corticotropin-releasing factor receptor antagonist (astressin; 100 µg/kg, *ip*), or the vasoactive intestinal peptide (VIP) receptor antagonist Lys^1^, Pro^2,5^, Arg^3,4^, Tyr^6^ (100 µg/kg, *ip*). Compared to sedentary rats, both the 2.5 and 5% exercise groups exhibited higher (P<0.05) values of blood lactate and fractional gastric dye recovery. Corticosterone and NO levels increased (P<0.05) in the 5% exercised rats. Pretreatment with astressin, VIP antagonist, atropine, L-NAME, and ODQ prevented the increase in gastric retention caused by exercise in rats. Acute exercise increased gastric retention, a phenomenon that appears to be mediated by the NO-cGMP pathway, CRF, and VIP receptors.

## Introduction

Regular physical activity has a notable impact on various physiological systems, such as cardiovascular (1) and neuromuscular (2) systems. Athletes, especially runners, report gut dysmotility during training and racing. Women, in particular, appear to suffer more commonly. Nearly half have loose stools, and nausea and vomiting frequently occur after hard runs. According to Gil et al. (3), 20 to 50% of the athletes that perform high-intensity physical exercise (particularly those of aerobic nature, such as cycling, marathons, and triathlons) present gastrointestinal complaints, including diarrhea and incontinence; rectal bleeding is also frequent (4). Runners usually take prophylactic medications to minimize some of these disorders. Upper digestive symptoms seem to occur more often in multisport events (e.g., triathlons) (5). Thus, physical exercise can be both beneficial and harmful for the gastrointestinal tract in a dose-effect relationship between its intensity and health (4).

We recently found that acute high-intensity exercise delayed the gastric emptying (GE) of a liquid test meal in awake male rats, a phenomenon that appears to be related to blood academia (6). A positive correlation was verified between blood lactate and fractional gastric dye recovery in exercised rats. The administration of an NH_4_Cl-containing meal in control rats induced metabolic acidosis and increased fractional gastric dye recovery to a similar degree as in the exercised group. Pretreatment with NaHCO_3_ corrected with lactacidemia and prevented the acute exercise-induced delay in GE (6). In addition, Chang and collaborators (2006) observed that lactate administration to sedentary and exercised rats delayed the GE of liquid *per se* (7).

Moreover, physical exercise has also been shown to lead to the release of several neurotransmitters and hormones that could influence gut motility (8). The role of vagal nerves on the gastric accommodation reflex and gastric emptying of liquids in mammals is well understood (9). The neurotransmitters nitric oxide (NO) and vasoactive intestinal peptide (VIP), found in the myenteric plexus, exert critical inhibitory roles on gut smooth muscle cells (10). High-intensity exercise raises blood levels of NO and VIP (11), but also promotes the release of corticosterone (12). Additionally, atropine has been shown to delay GE (13). We hypothesized that the acute exercise-induced delay in GE involves vagal nerves that are associated with the corticotropin-releasing factor (CRF) and VIP, as well as cGMP-NO/VIP pathways. Thus, the present study aimed to evaluate the effect of cholinergic, nitrergic, and VIPergic pathways as well as CRF receptors in the present phenomenon.

## Material and Methods

### Ethical approval

The experimental protocol and animal handling followed the recommendations of the Guide for the Care and Use of Laboratory Animals (US National Institutes of Health, 1996). All of the procedures were reviewed by and had prior approval of the Animal Ethics Committee (UNINTA, Brazil, protocol No. 2014.03.002-P).

### Animals

Male Wistar rats (250–300 g) were obtained from the animal housing center of the Federal University of Ceará and kept under controlled conditions of stable temperature (28°C±1°C) and a 12 h/12 h light/dark cycle, with free access to food and water. The animals were allocated into different subgroups (n=5–12).

### Acute exercise protocol

Acute exercise was performed according to a previously described protocol (14) that was adapted by our group (6) (Supplementary Table S1). All of the rats initially underwent an adaptation period that consisted of swimming in a cylindrical tank (50 cm deep, 60 cm in diameter) filled with warm water (30°C±1°C). In order to minimize animal stress, the free-swimming time interval increased in a stepwise fashion from the first to the last session, respectively: day 1 (10 min), day 2 (20 min), day 3 (30 min), day 4 (40 min), and day 5 (40 min). Each session was conducted once daily at 12:00 PM. The animals were dried after each session with an absorbent towel and blow dryer and returned to their cages. One day after the last exercise session, all of the rats were kept individually in metabolic cages and fasted for 18 h with free access to an oral rehydration solution consisting of 75 mM Na^+^, 65 mM Cl^-^, 20 mM K^+^, 75 mM glucose, and 10 mM citrate (7). After 24 h, they were divided into two groups, subjected or not (sedentary control group) to another 15-min acute swimming exercise session, during which the rats performed the exercise with a load equivalent to 2.5 or 5% of their body weight (2.5% and 5% exercised groups). Loading was achieved by tying fishing sinker weights with an elastic string around the animal's chest. The sedentary rats remained for 15 min without the additional load in a nearly empty cylindrical tank that contained water at a depth of only 5.0 cm. Immediately after the acute exercise or sedentary protocols, a drop of blood was drawn from the tail vein of each rat to measure lactic acid concentrations using an automatic analyzer (Accutrend Plus, Roche®, Portugal). Lactate analysis was performed to estimate exercise intensity.

### Body composition assessment

In order to assess body composition, the bioimpedance spectroscopy (BIS) method (ImpediMED®, Australia/New Zealand) was employed, according to a previous report (15). A separate group of rats was randomly subjected to either the sedentary or exercise (2.5% or 5%) protocol, as formerly described. At the end of the session, they were anesthetized with tribromoethanol (250 mg/kg, *ip*) and placed in supine position on a flat surface for the insertion of four electrodes via hypodermic needles (two in the head and two in the tail). The electrodes were then connected to the BIS device to assess fat mass (FM; in g or percentage), fat-free mass (FFM; in g or percentage), total body water (TBW; in mL or percentage), extracellular fluid (ECF; in mL or percentage), intracellular fluid (ICF; in mL or percentage), and body mass index (BMI; in g/cm^2^).

### Gastric emptying assessment

One day after the adaptation period, the rats were subjected to 18 h of fasting (with free access to the oral rehydration solution) and either the sedentary or exercise (2.5% or 5%) protocols. Next, they were gavage-fed with a liquid test meal that consisted of 1.5 mL of 50 mg/mL phenol red in a 5% glucose solution. After a 5, 10, or 20 min postprandial interval, the rats in the sedentary and 5% exercised groups were euthanized by a thiopental overdose (100 mg/kg, *ip*). The rats in the 2.5% exercised group were euthanized 10 min postprandially. Fractional gastric dye recovery was assayed according to Reynell and Spray (14), a technique that was further adapted by our laboratory (6). After surgical exeresis, the gut was quickly ligated at the esophagus, duodenum, and terminal ileum, and divided into consecutive segments: stomach, proximal small intestine (∼40%), mid small intestine (∼30%), and distal small intestine (∼30%). The volume of each portion was evaluated by placement into a graduated cylinder that contained 100 mL of 0.1 N NaOH solution. The segments were then cut and homogenized with a mixer for 30 s. The suspension was allowed to settle for 20 min, and 10 mL of the supernatant was centrifuged at 1229 *g* for 10 min at 25±2°C. Proteins were precipitated with 0.5 mL of 20% trichloroacetic acid solution. After centrifugation, 3.0 mL of the supernatant was added to 4.0 mL of 0.5 N NaOH, and the samples were read using a spectrophotometer at 560 nm (BIOCHROM Ltd., England) to construct dilution curves by plotting the dye concentrations against optical densities (ODs). The linear coefficient (α) of the dilution curve defined the solution concentration (C=OD) and the amount of phenol red (m) that was recovered from each segment (m=C × volume). Fractional gastric dye recovery values were calculated as follows: gastric dye recovery (%) = 1 – (amount of phenol red recovered in the stomach / total amount of phenol red recovered from all segments) × 100.

In order to investigate the neurohumoral pathways involved in acute exercise-induced GE delay, separate groups of rats were subjected to the same previously described procedures. On the day of the experiment, the animals were randomly treated with vehicle (0.9% NaCl, 0.1 mL/100g, *ip*) or one of the following agents: the muscarinic receptor antagonist (atropine, 1.0 mg/kg, *ip*) (17), the NO non-selective inhibitor Nω-nitro-L-arginine methyl ester hydrochloride (L-NAME; 10 mg/kg, *ip*) (18), the cGMP selective inhibitor 1H-(1,2,4)oxadiazole[4,3-a]quinoxalin-1-one (ODQ; 5.0 mg/kg, *ip*) (19), the i-NOS non-selective inhibitor (aminoguanidine 10 mg/kg, *ip*) (20), the CRF receptor antagonist (astressin 100 µg/kg, *ip*) (21), or the VIP receptor antagonist Lys^1^, Pro^2,5^, Arg^3,4^, Tyr^6^ (100 µg/kg, *ip*) (19). Thirty minutes after pretreatment, the rats were subjected to the formerly specified sedentary or acute exercise protocols. They then received the liquid test meal and were euthanized 30 min postprandially by a thiopental overdose (100 mg/kg, *ip*) to calculate fractional gastric dye recovery, as previously described.

### Nitric oxide and corticosterone analysis

Separate groups of rats were subjected to either the sedentary or 5% acute exercise protocols, followed immediately by euthanasia and decapitation. In order to evaluate plasma NO levels, blood samples were collected, and the serum was stored at –20°C. The plasma levels of NO were measured as light emissions produced by the reaction between NO and ozone (O_3_), detected by chemiluminescence (22). For corticosterone evaluation, blood was collected and stored in frozen plastic tubes that contained heparin (10 μL per mL of blood). Plasma was obtained after centrifugation at 1411 *g* for 20 min at 4°C and stored at –20°C until the specific extraction and immunoassay procedures were initiated. Corticosterone was extracted from 25 μL of plasma with 1.0 mL of ethanol using Sep-Pak C-18 cartridges (Waters, USA). Corticosterone measurements were performed using specific radioimmunoassay techniques, as previously described (23). All of the measurements were conducted in duplicate in the same assay. The sensitivity of the assay and the intra- and inter-assay coefficients of variation were 0.4 µg/dL and 8.0–10.6%, respectively, for corticosterone.

### Statistical analysis

Initially, the data were analyzed for normal distribution by the Shapiro-Wilk normality test. Data analysis was conducted using the GraphPad Prism software version 6.0 (USA) and, for comparisons between two groups, the Student's *t*-test for unpaired data was employed. For comparisons between three or more groups, one-way analysis of variance (ANOVA) was performed, followed by Tukey's *post hoc* test. Two-way analysis of variance (ANOVA) followed by Tukey's *post hoc* test was carried out to compare the interaction of more than two variables between groups. Data are reported as means±SD. P<0.05 was considered statistically significant.

## Results

The mean values of body composition indices assessed by the BIS method in the sedentary and acute exercise (2.5% and 5%) rats are shown in Table 1. No significant difference was found between groups regarding the following parameters: TBW, ECF, ICF, FFM, FM, or BMI.

**Table 1:** Evaluation of body composition by the bioimpedance spectroscopy (BIS) method in rats that were subjected to the sedentary or acute exercise (2.5% or 5%) protocols.

	Sedentary	2.5% Acute exercise		5% Acute exercise	
TBW					
mL	111.1±15.7	112.5±9.7	n.s.	102.7±8.0	n.s.
%	44.8±8.1	43.5±3.1	n.s.	42.3±3.9	n.s.
ECF					
mL	53.2±5.2	56.2±6.2	n.s.	49.8±2.5	n.s.
%	48.8±4.5	49.6±6.2	n.s.	48.6±2.1	n.s.
ICF					
mL	57.8±11.4	57.8±10.5	n.s.	52.9±5.5	n.s.
%	51.2±4.5	60.4±10.4	n.s.	52.8±0.9	n.s.
FFM					
g	151.8±21.5	155.8±13.1	n.s.	140.3±11	n.s.
%	61.2±11.0	59.4±4.2	n.s.	57.7±5.3	n.s.
FM					
g	95.6±19.0	105.8±6.8	n.s.	103.1±15.3	n.s.
%	38.7±11.0	40.5±4.2	n.s.	42.2±5.3	n.s.
BMI					
g/cm2	8.7±0.5	9.1±0.5	n.s.	9.2±0.7	n.s.

TBW: total body water; ECF: extracellular fluid; ICF: intracellular fluid; FFM: fat-free mass; FM: fat mass; BMI: body mass index. The data are reported as means±SD and were analyzed by ANOVA followed by the Tukey test. n.s.: not statistically significant.

**Figure 1:**
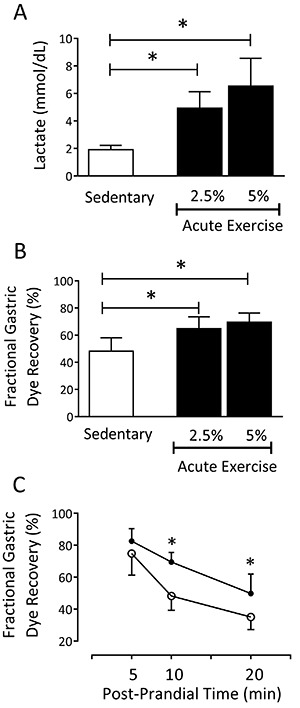
Comparison of blood lactate concentrations (*A*) and fractional gastric dye recovery (*B*) in sedentary and acute exercised (2.5% or 5%) rats at 10 min postprandially. The data are reported as means±SD. *P*<*0.05, sedentary *vs* acute exercise (2.5% or 5%; ANOVA followed by Tukey's *post hoc* test). *C*, gastric retention curve over time in sedentary (clear circles) and acute exercise (5%) rats (black circles) at 5, 10, and 20 min postprandially. The data are reported as means±SD. *P*<*0.05, 5% acute exercise compared to sedentary at 10 and 20 min postprandially (two-way ANOVA followed by the Tukey's *post hoc* test).

As shown in Figure 1, the acute exercise (2.5% and 5%) protocols significantly increased (P=0.0001) plasma lactate concentrations compared to the sedentary rats (sedentary: 1.9±0.3 mmol/dL, n=7 *vs* 2.5% exercise: 4.9±1.1 mmol/dL, n=7 *vs* 5% exercise: 6.5±2.0 mmol/dL, n=11), as well as the gastric retention of the liquid test meal at 10 min postprandially (sedentary: 48.2±9.8%, n=12 *vs* 2.5% exercise: 64.7±8.7%, n=8, and 5% exercise: 69.5±6.8%, n=12; Figure 1B). According to Figure 1C, exercise induced GE delay of the liquid test meal in awake rats. At 5 min postprandially, no significant difference in mean values of fractional gastric dye recovery was observed between sedentary and 5% exercised rats (sedentary: 74.8±12.1%, n=10 *vs* 5% exercise: 82.1± 7.3%, n=10). However, at 10 min postprandially, a significant increase (P=0.0001) in the parameter (sedentary: 48.2±9.8%, n=10 *vs* 5% exercise: 69.5±6.8%, n=10) was noted, which persisted until 20 min postprandially (sedentary: 35.0±6.5%, n=10 *vs* 5% exercise: 49.8± 10.8%, n=10).

**Figure 2:**
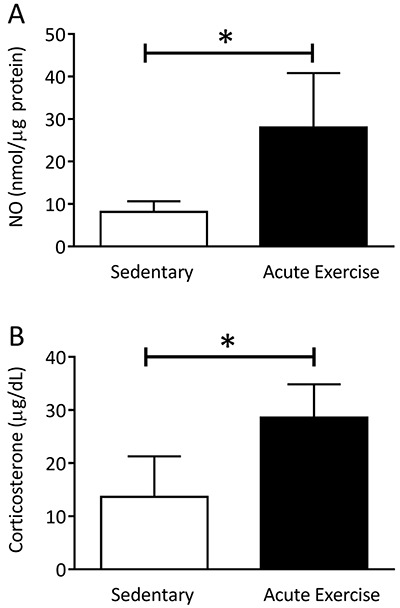
Comparison of plasma levels of nitric oxide (*A*) and corticosterone (*B*) in rats subjected to the sedentary or acute exercise (5%) protocols. The data are reported as means±SD. *P<0.05, sedentary *vs* 5% acute exercise (Student's *t*-test).

As shown in Figure 2, the 5% acute exercise protocol significantly increased (P = 0.0002) plasma NO levels compared to the sedentary rats (sedentary: 8.1±2.4 nmol/µg protein), n=10 *vs* 5% exercise: 28.0±12.7 nmol/µg protein, n=9; Figure 2A), as well as plasma corticosterone levels (sedentary: 13.6±7.6 mg/dL, n=9 *vs* 5% exercise: 28.5± 6.2 mg/dL, n=10; Figure 2B) (P=0.0002).

**Figure 3:**
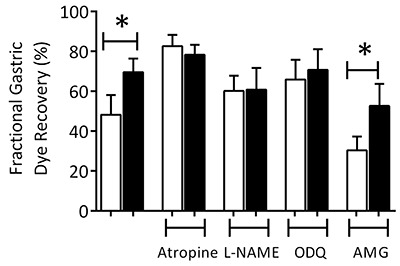
Neurohumoral mechanisms of acute exercise-induced gastric emptying delay. The figure shows the effects of pretreatment with atropine (1.0 mg/kg, *ip*), L-NAME (10 mg/kg, *ip*), ODQ (5.0 mg/kg, *ip*), and aminoguanidine (AMG, 10 mg/kg, *ip*) on gastric retention of a liquid test meal in rats that were subjected to the sedentary (white bars) or acute exercise (5%) (black bars) protocols. The data are reported as means±SD. *P<0.05 (ANOVA followed by the Tukey's test).

When evaluating the role of neuro-autonomic pathways in the exercise-induced GE delay, no difference was observed between the fractional gastric dye recovery values of atropine, L-NAME, and ODQ pretreated rats, subjected or not to acute exercise [82.6±5.6% (N=5) *vs* 78.3±4.9% (N=5), 60.7±10.9% (N=9) *vs* 60.1±7.6% (N=8), and 65.9±9.8% (N=8) *vs* 70.7±10.3% (N=8)], respectively. In contrast, in the aminoguanidine pretreated group, the rats subjected to 5% exercise showed increased gastric recovery values compared to the respective controls (30.3%±6.9%, n=5 *vs* 52.6%±11.1%, n=5), as shown in Figure 3.

**Figure 4:**
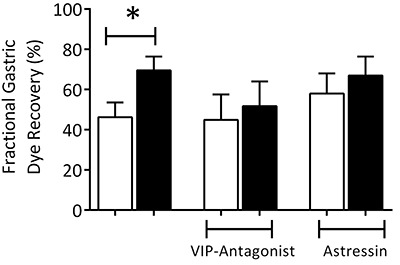
Neurohumoral mechanisms of acute exercise-induced gastric emptying delay. The figure shows the effects of pretreatment with the vasoactive intestinal peptide (VIP) antagonist Lys^1^, Pro^2,5^, Arg^3,4^, Tyr^6^ (100 μg/kg, *ip*) and astressin (100 μg/kg, *ip*) on gastric retention of a liquid test meal in rats that were subjected to the sedentary (white bars) or acute exercise (5%) (black bars) protocols. The data are reported as mean±SD. *P*<*0.05 (ANOVA followed by the Tukey's test).

In Figure 4, it can be noted that VIPergic pathways and the CRF receptors were also involved in exercise-induced GE delay in awake rats. No difference in mean fractional gastric dye recovery was observed in VIP antagonist-pretreated rats subjected or not to 5% exercise (44.8± 12.6%, n=5 *vs* 51.7±12.2%, n=5). Regarding the involvement of CRF receptors in this phenomenon, changes were not observed in mean fractional gastric dye recovery by astressin-pretreated rats subjected or not to acute exercise (66.9±9.3%, n=7, *vs* 57.9±9.9%, n=6).

## Discussion

In the present study, high-intensity acute swimming did not affect body composition but did delay the GE of a liquid meal in awake rats, a phenomenon that appears to involve the NO-cGMP and cholinergic pathways, as well as VIP and CRF receptors.

The body composition of sedentary and exercised rats was assessed using the BIS method. Chronic exercise alters the body composition of mammals by promoting fat tissue loss and increasing protein synthesis and muscle mass, a process that varies according to the nature of the physical activity (24). Exercise may also alter body water content by inducing dehydration. We previously showed that acute changes in blood volume modify the rate of GE of liquid test meal in rats (25). However, herein, acute exercise with 2.5 and 5% load did not alter body composition. Thus, it can be suggested that changes in body indices require chronic training (26).

Also, we assessed the GE rate using a simple and reliable technique (i.e., dye dilution); however, this method can be influenced by pH, which may induce false positivity (16). Acute exercise does not promote changes in gastric acid secretion (4). We recently showed that pretreatment with omeprazole did not influence the GE delay induced by acute exercise (5).

Both acute and chronic exercise promotes physiological adaptations. Many parameters can be employed to assess these adaptations, including maximum oxygen consumption (VO_2_max) (2) and blood lactate (27). In the present investigation, the lactate threshold was used to prescribe the intensity of acute exercise, as proposed in previous studies (2). Thus, we opted for acute exercise for 15 min with a 2.5 or 5% body weight overload, which characterizes high-intensity activity, with increased levels of blood lactate. Acute exercise protocols stimulate anaerobic lactic metabolism in skeletal muscle cells, disrupt the acid-base balance, and lower blood pH, thus delaying the GE of liquid test meals in awake rats (6). In the present study, acute exercise for 15 min against an overload equivalent to 5% of body weight increased fractional gastric dye recovery. Postprandially, this phenomenon became evident at 5 min, was statistically significant at 10 min, and persisted for at least 20 min. In order to further determine the mechanisms involved in this phenomenon, distinct groups of rats subjected or not to acute exercise (5% overload) were evaluated at 10 min postprandially.

Several peptides and hormones present in the enteroinsular axis play significant roles in body homeostasis, and their secretion is altered by physical exercise (28). In this sense, the metabolic effects of corticosterone are modulated by internal and external environmental conditions (29). In our study, we show that acute exercise for 15 min against an overload of 5% of body weight increased serum corticosterone levels. Therefore, high-intensity exercise appeared to raise corticosterone secretion *per se*. The obtained plasma corticosterone results from sedentary rats are similar to those described by other authors with rats in hydromineral balance (22). Radahmadi et al. (28) previously reported a similar increase in serum corticosterone levels in trained rats. Intracerebroventricular injection of CRF also delayed GE, and this effect was abolished by peripheral administration of CRF receptor antagonists (29). In fact, our data show that the CRF system plays a critical role in delaying GE in exercised rats, in which pretreatment with the CRF receptor antagonist astressin prevented the acute exercise-induced increase in fractional gastric dye recovery (30).

The gastroduodenal flow of liquid meals in mammals is determined by a complex process. The present exercise-induced GE delay may have resulted from an increment in gastric compliance, decrease in antral contractility, or increase in pyloric or duodenal resistance (6,16). The gastric emptying rate is subjected to the influence of sympathetic and parasympathetic neural pathways. Efferent fibers of vagal nerves maintain the tonic activity of smooth muscle cells of the proximal stomach, driving the gastric fundus tone during fasting. During and immediately after meal ingestion, the proximal stomach relaxes via non-adrenergic and non-cholinergic pathways, enabling an increase in gastric volume without a concomitant rise in intraluminal pressure (31). In turn, the stimulation of muscarinic acetylcholine receptors has excitatory effects on the gastric tonus, and atropine impairs the gastric accommodation reflex in rats, as reported by Verschueren et al. (31). In the present study, atropine treatment prevented the exercise-induced increased gastric retention of the liquid test meal.

In our study, we suggested that the muscarinic antagonist atropine inhibits the excitatory parasympathetic pathway, fosters gastric relaxation, and increases gastric retention in exercised rats (13). Acute exercise promotes the release of several vasodilator agents such as nitric oxide, prostaglandins, and the endothelial-derived hyperpolarizing factor (32,33). Recently, physical activity was shown to elicit hyperemia in skeletal musculature via recruitment of a NO-cGMP pathway (34) in healthy subjects (35). Herein, the 15-min acute exercise against 5% of body weight increased the circulating levels of NO in rats, as reported in previous studies (36). Indeed, Lefebvre et al. showed that L-NAME prevented intra-gastric pressure reduction during vagal nerve stimulation in anesthetized rats, indicating that NO release is crucial for this response, given that the neurotransmitter is an essential mediator in gastric motility (36).

In addition, NO also acts as an inhibitory neurotransmitter in the gut and is involved in smooth muscle relaxation and motility (37). The role of a nitrergic-cGMP pathway in the present phenomenon is indicated by the fact that both pretreatments with L-NAME, a NO synthase inhibitor, or the cGMP inhibitor ODQ prevented the increase in gastric retention verified in acutely trained rats.

On the other hand, it is known that decreased gastric emptying may occur during inflammatory processes (35). However, in the present study, pretreatment with aminoguanidine, a non-specific i-NOS inhibitor, failed to avert the increment in gastric retention, indicating that the effect is probably not inflammatory.

Another critical non-adrenergic and non-cholinergic (NANC) inhibitory agent in the gut is VIP, which is found predominantly in myenteric motor neurons (19). The agent is released from gut preparations upon electrical field stimulation of intrinsic nerves. Exogenous VIP mimics the NANC relaxation of gut smooth muscles, reducing VIP antagonism by specific antibodies (19). VIP activates adenylate cyclase through G-protein-coupled receptors in gut smooth muscle cells, elevating intracellular levels of cAMP and eliciting smooth muscle relaxation. According to MacLaren et al. (38), acute exercise, such as endurance exercise and forced swimming, raises blood VIP levels. In addition to eliciting splanchnic hyperemia, VIP also stimulates lipolysis, glycogenolysis, and gluconeogenesis, favoring energy mobilization during physical activity (28). In the present study, pretreatment with a VIP receptor antagonist inhibited the acute exercise-induced GE delay in awake rats. Thus, physical exercise increases VIP blood levels, which may relax gastric musculature and inhibit the gastric emptying rate.

Exercise intensity seems to be a fundamental criterion in evaluating the rate of gastric emptying, in which greater intensity causes the most significant disturbance in gastric mobility. Slower emptying with exercise at higher intensities and longer duration has been observed; however, the impact of acute exercise is not clear. Considering that physical activity promotes a potent sympathetic impulse, it is believed that sympathetic activation may delay gastric emptying in response to stress (39).

In addition, we recently showed that pharmacomechanical stimulation induced by cholinergic signaling through muscarinic receptors is affected by extracellular acidosis in isolated preparations of rat gastric fundus (40). This phenomenon could also be involved in the exercise-induced gastric emptying delay in rats (i.e., a condition that causes lactate-derived extracellular acidification) (7). Therefore, we hypothesize that acidosis may selectively interfere with the Gq/11 protein-phospholipase C signaling cascade, whereas signaling pathways that recruit voltage-operated Ca^2+^ channels remain functionally preserved.

Although exciting, the present findings cannot necessarily be extrapolated to athletic conditioning and performance training. Physical exercise is often accompanied by the ingestion of beverages, and their composition, volume, and energy density can alter the GE rate, which could influence several factors, such as exercise intensity and training conditions (39).

In conclusion, the present results suggest that acute high-intensity swimming delays the GE of liquid test meals in awake rats, a phenomenon that involves the NO-cGMP pathway and VIP and CRF receptors.

## Supplementary Material

Click here to view [pdf].
